# Effects of a proprioceptive focal stimulation (Equistasi®) on reducing the biomechanical risk factors associated with ACL injury in female footballers

**DOI:** 10.3389/fspor.2023.1134702

**Published:** 2023-07-14

**Authors:** Fabiola Spolaor, Annamaria Guiotto, Alfredo Ciniglio, Federica Cibin, Zimi Sawacha

**Affiliations:** ^1^ Department of Information Engineering, University of Padova, Padova, Italy; ^2^ BBSoF Srl, Padova, Italy; ^3^ Department of Medicine, University of Padova, Padova, Italy

**Keywords:** ACL injury prevention, proprioceptive mechanical focal stimuli, sidestep cutting maneuver, biomechanical risk factors, football player

## Abstract

**Introduction:**

Football presents a high rate of lower limb injuries and high incidence of Anterior Cruciate Ligament (ACL) rupture, especially in women. Due to this there is the need to optimize current prevention programs. This study aims to verify the possibility to reduce the biomechanical risk factors associated with ACL injury, through the application of proprioceptive stimulation by means of the Equistasi® device.

**Methods:**

Ten elite female footballers were enrolled and received the device for 4 weeks (5 days/week, 1h/day). Athletes were assessed directly on-field at four time points: T0 and T1 (evaluation without and with the device), T2 (after 2 weeks), T4 (after 4 weeks) while performing two different tasks: Romberg Test, and four sidestep cutting maneuvers bilaterally. Seven video cameras synchronized with a plantar pressure system were used, thirty double colored tapes were applied on anatomical landmarks, and three dimensional coordinates reconstructed. Vertical ground reaction forces and center of pressure data were extracted from the plantar pressure insoles. Hip, knee, and ankle flexion-extension angles and moments were computed as well as abd-adduction joint torques. From the Romberg Test both center of pressure descriptive variables and frequency analysis parameters were extracted. Each variable was compared among the different time frames, T1, T2 and T4, through Friedman Test for non-parametric repeated measures (p<0.05); Wilcoxon Signed Rank Test was used for comparing variables between T0 and T1 (p<0.05) and across the different time frames as follows: T1–T2, T2–T4 and T1–T4.

**Results:**

Statistically significant differences in both posturographic and biomechanical variables between the assessment at T0 and T1 were detected. Reduced hip and knee abduction torques were revealed in association with reduced both ground reaction forces and ankle dorsiflexion torque from T1 up to T4.

**Discussion:**

The proprioceptive stimuli showed to have the potential to improve cutting biomechanics mainly with respect to the ligament and quadriceps dominance theories. Results of the present study, even if preliminary and on a small sample size, could be considered promising towards the inclusion of proprioceptive training in injury prevention programs.

## 1. Introduction

According to FIFA, football is the world's most popular sport with more than 265 million players (1), and female professional, semi-professional, and community football is one of the fastest growing sport worldwide. Along with its popularity, football accounts for a higher rate of lower-limb injuries accompanied by a higher incidence of anterior cruciate ligament (ACL) rupture and associated burden than do other sports (2–5). Females have a higher risk of concussion and ankle injuries, and the risk of serious knee injuries is at least double when compared with men; meanwhile, men present greater risk of hamstring and groin injuries (6–12). In particular, ACL injury rate is higher during match play compared with training, and in cutting and pivoting sports (13) compared to other sports. In this scenario, non-contact ACL injuries, that typically occur during high-impact tasks such as decelerations, landing, and cutting represent one of the most devastating injuries, accounting for 70% of ACL annual injuries with an incidence rate of 0.42 per 1,000 player-hours (14). The long return to play period (6–24 months) and an increased risk in future development of osteoarthritis represent the associated burdens. It should be mentioned that injury, fear of injury, and lack of physical skills or strength represent the current barriers to sport and physical activity participation in females (15). All these reasons support the introduction of new methodologies to prevent or reduce the risk of injury. Nowadays, a large variety of functional screening tools are adopted during pre-season tests to assess athletes’ readiness to compete and to establish a baseline measurement at the beginning of a rehabilitation program. These tests allow tracking athletes’ progress and assisting in return to play decisions, along with identifying specific movement faults, muscle weakness, imbalances. In terms of functional evaluation, the most commonly used methods are the Functional Movement Screen, the Star Excursion Balance Test, the Y Balance Test, the Drop Jump Screening Test, the Landing Error Scoring System, and the Tuck Jump Analysis (16): they imply visual scoring of different movements as well as pain, and the best score is associated with the ability to correctly complete the movement without compensation. In considering the possible causes of ACL rupture, non-contact ACL injuries have been reported as the most frequent that commonly occur when landing from a jump, during cutting maneuvers, or sudden decelerations (17). There is a plethora of studies reporting about the association between ACL rupture and a poor landing mechanism (18): with the knee in a valgus position and the femur in adduction and internal rotation; with the knee in an extended position and with excessive quadriceps activation relative to the hamstrings; with deficits in trunk control; and with large leg-to-leg asymmetries (18). These considerations are at the basis of the standard functional tasks currently adopted in the pre-season tests (19). However, the large number of ACL injuries still reported annually (80,000 per year in the United States alone) (20) poses the need for screening athletes through other tasks that can better portray the multifactorial mechanism of ACL rupture. Among the possible ones, cutting maneuvers has recently attracted a large attention as they represent the primary cause of non-contact injuries in cutting dominant sports (i.e., rugby, American football, handball, basketball, football) (14, 21). In this context, the injury mechanisms that have been most commonly described include frontal plane trunk misalignment with respect to the cutting direction, internally rotated hip, valgus knee alignment at loading response, an excessive extended knee, hip abduction, increased foot progression angle, rearfoot landing, and excessive lateral foot-plant distances. All these conditions evoke the greatest multiplanar knee joint loading (14, 22). In particular, when females perform cutting maneuvers, landing with an extended hip and knee, with a valgus knee alignment, an internal rotation of the tibia, and a pronated foot (i.e., “position of no return”) have been indicated as the “high-risk postures” (23). We should further consider that, when ACL is placed under stress, hamstrings are activated by both a fast-to-respond reﬂex arc from ACL mechanoreceptors to the hamstrings, and a secondary reflex arc from mechanoreceptors in the muscle or joint capsule. The first one allows the hamstrings to act as a torque regulator “on demand” during ligament overloading, thus supporting in maintaining joint stability. The second one, thanks to a longer response time than the first one, and an inhibitory input to the quadriceps, provides activation of the hamstrings to assist knee instability conditions (24). However, elite athletes are subjected to nonphysiologically large forces coupled with fast rates of load applied to the knee, which do not allow these two reflex arcs to respond fast enough (18). This is one of the main reasons why thigh muscle exercises and strength improvements may not be sufficient to provide the necessary support to reduce incidences of instability during high-impact tasks (i.e., decelerations, landing, and changes of direction). It is in this general framework that the inclusion of proprioceptive training finds its applicability in prevention and rehabilitation programs (25). Given the importance of motor control in restoring athletes motor function after injury, therapies have been recently proposed targeting at training the proprioceptive system to achieve coordinated sensory–motor patterns in human movements. On the one hand, we can find neuromuscular and proprioceptive training exercise (26) and, on the other, vibrations applied to muscles or tendons that can induce different effects depending on the vibrated muscle, the sensory context, and the task. In the study by Melnyk et al. (27) whole-body vibrations were applied on stretch reflexes involved in knee joint control as ACL injuries prevention treatment. Results of the study showed improved knee stability and reduced anterior tibial displacement upon shock provocation (28). Another non-invasive therapeutic modality commonly used is transcutaneous electrical nerve stimulation; this technique is based on the stimulation of the large diameter mechanosensitive afferent nerve fibers in the skin but it is mostly used in pain control (29). Recently, a neurorehabilitation device, Equistasi®, based on focal mechanical vibrations (30) has been applied to modify cyclists’ pedaling posture (31), and this was reflected in a better distribution of the pressure on the saddle of a group of amateur cyclists. The device was originally adopted for therapeutic purposes in Parkinson's disease targeting at improving trunk sway (32) and gait (33). The Equistasi® device, once applied on specific sites of the body (i.e., muscles, trigger points), self-generates focal mechanical vibrations that interacts with the mechanoreceptors, the Golgi tendon organs, and the neuromuscular spindles. To the best of the authors’ knowledge, vibrations have been applied for ACL injury prevention purposes as a whole-body solution (27, 28); therefore, the aim of the present study is to evaluate the impact of providing a proprioceptive stimulus through the Equistasi® device on the biomechanical risk factors associated with ACL injury. For this purpose, a biomechanical evaluation was carried out directly on the field and involved a group of elite female football players performing a series of sidestep cutting maneuvers. This task was selected as it is similar to the sport gestures involved in competitions, and accounts for the majority of non-contact injuries. From a biomechanical point of view, externally applied knee abduction, internal rotation, flexion-extension torques, knee flexion angle, and anterior tibial shear forces have all been identified as the currently agreed biomarkers of non-contact ACL injury risk (14). Furthermore females have been reported to use both an increased knee valgus kinematics and decreased hamstrings-to-quadriceps peak torque ratio compared to males (17). Of these biomarkers, in the present study, lower-limb joint angles and torques were considered, and a female elite team was selected, due to the higher rate of incidence of ACL rupture in females than males. The main hypothesis of the present study is that by improving proprioception through the use of the Equistasi® device, the multiplanar loading condition associated with increased ACL strain could be reduced, and consequently this could be reflected in the performance of a high-risk task such as sidestep cutting maneuver. In order to verify the effective elicitation of the somatosensory system, and its link with motor control, through the application of the vibratory stimulus, the Romberg test was performed similar to previous studies aiming at restoring balance and motor control in individuals with Parkinson's disease (32, 33).

## 2. Material and methods

### 2.1. Participants

Ten female footballers (Table 1) competing in the first Italian division were enrolled, after signing informed consent to participate in the study. The protocol was approved by an ethics committee (CAR 107/2021). Inclusion (exclusion) criteria were as follows: no history (history) of previous severe injuries in the 6 months preceding the study; no identified restrictions (having specific restrictions) to sports practice in agreement with the team sport medicine physicians, and no previous ACL rupture. As no data from similar studies were available, *a posteriori* power analysis (G * Power 3.1) was performed, which indicated a power (1 − β err prob) of 0.9 for a sample of 10 subjects based on the posturographic variables (34).

**Table 1:** Demographic data of the participants involved.

Age (years)	Weight (kg)	Height (m)	BMI (kg/m^2^)	Shoe size	Dominant laterality	Role
Mean (SD)	Mean (SD)	Mean (SD)	Mean (SD)	Mean (SD)		
25.30 (3.65)	58.38 (7.25)	1.66 (0.06)	21.23 (1.71)	38.45 (1.28)	Right: 80% Left: 20%	Defender: 50% Midfielder: 10% Striker: 40%

### 2.2. Equistasi® device

Equistasi® is a wearable and innovative medical device, approved by the Ministry of Health with the CNN product code number 342,575 and COP product code number 342,577. It is based on a vibrational technology: it self-generates focal mechanical vibrations at a non-constant frequency of about 9,000 Hz, within the limits imposed by Legislative Decree 81/08 (30).

Equistasi® device has the following characteristics:

• It is applied as a regular bandage strip and can be worn during motor activities;
• It is 1 cm × 2 cm in size and has a weight of 0.17 g;
• It can be reused several times; and
• Other than general wear, it does not expire.

Equistasi® is composed exclusively of nanotechnology fibers and does not contain any pharmacological elements. The stimuli generated by the vibrations transmits the information to the central nervous system; once applied on the affected muscular areas, the focal mechanical vibrations interact with the mechanoreceptors, the Golgi tendon organs, and the neuromuscular spindles (35).

### 2.3. Study design

**Figure 1:**
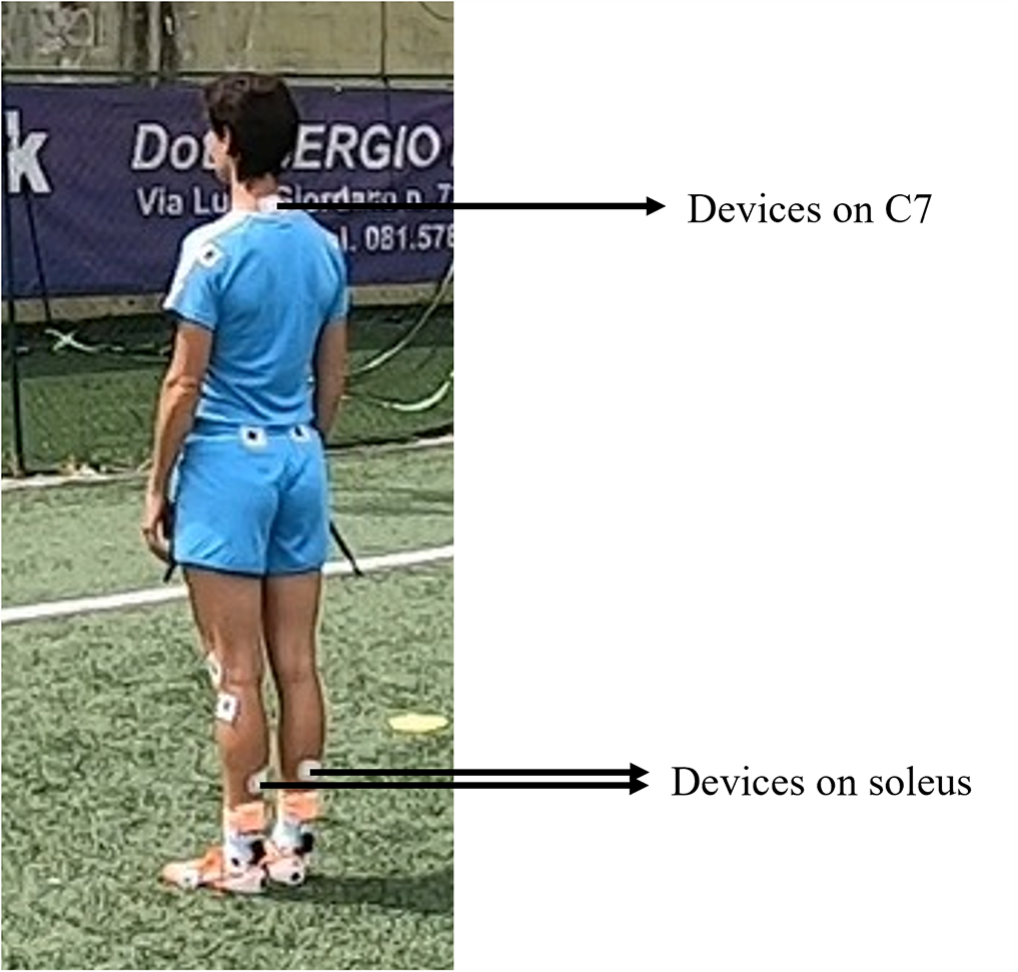
Equistasi® device placement.

All the athletes received the Equistasi® device for 4 weeks. The device was applied to the skin in the standard configuration as follows (36, 37): one on the seventh cervical vertebra and two on each soleus muscle (Figure 1). The device was worn for 1 h/day, 5 days/week (36, 37). The consistency of the study was monitored by telephone contacts with team official doctors and athletes. The athletes were assessed at four time points:

• T0: first assessment without wearing the Equistasi® device;
• T1: first assessment while wearing Equistasi® device;
• T2: after 2 weeks of application of the device; and
• T4: after 4 weeks of application of the device.

### 2.4. Instrumental protocol and data collection

All data were acquired at the team's site through seven commercial cameras (four GoPro Hero 7 and three GoPro Hero 3, 1080 × 1920/4 × 3k pixel resolution, 30/240 fps) synchronized with a wireless plantar pressure system (Blue Insole insoles, FGP s.r.l. and Motux S.r.l. software, 214 resistive sensors, 200 Hz). Markers, made with double-colored tape, were applied on each subject according to the IORgait marker set (31, 38). All the data were synchronized in post processing through self-developed Matlab codes (Matlab R2021). All the subject performed two different tasks on natural grass field as follows:

Romberg test: participants were required to stand for 60 s in both eyes open (EO) and eyes closed (EC) conditions, with their arms along the body and the feet 30° apart (assured by a cardboard triangle) (39, 40). The rationale behind this test was first of all to assess the impact of the device on the proprioceptive system (i.e., comparison between T0 and T1, with special focus on the frequencies analysis, see Section 2.5), and second to evaluate the possible impact on athletes’ balance over the time (i.e., all posturographic variables in the following comparisons: T1–T2, T2–T4).

**Figure 2:**
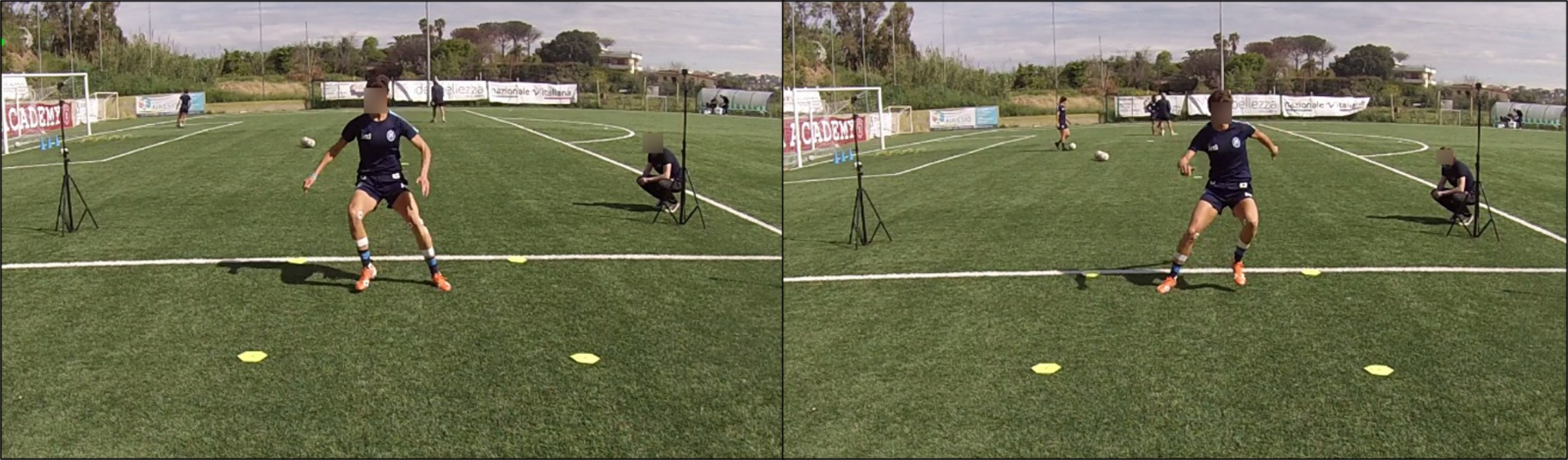
An athlete during the sidestep cutting maneuver execution.

Sidestep cutting maneuvers: all the athletes performed four sidestep cutting maneuvers (two left side and two right side) starting from a 5 m sprint; the direction of the sidestep cutting maneuver was randomly assigned by the personal trainer during task execution (see Figure 2).

### 2.5. Data analysis and variables

Once acquired, the video sequences were processed and three-dimensional anatomical landmark coordinates extracted through “Track On Field” software (BBSoF S.r.l.), validated in the study by Guiotto et al. (16). From the plantar pressure insoles, the center of pressure (COP) was extracted together with the vertical ground reaction forces. The data were filtered with similar filters for kinematic and kinetic parameters when involved in the calculation of joint torques, according to Derrick et al. (41). Anatomical landmark trajectories were filtered with a low pass Butterworth filter (6 Hz cut off frequency) and the plantar pressure data were filtered with a fourth-order low pass Butterworth filter (6 Hz cut of frequency) when the ground reaction forces were extracted and with a fifth-order low pass Butterworth filter (7 Hz cut of frequency) when the COP was assessed (16).

From the instrumented Romberg test (42), the following variables were extracted:

• The COP displacement [e.g., COP path, COP anterior posterior path (Path Z), COP medial lateral path (Path X)];
• COP velocity (Vel), COPVel along both the *x* and *z* axis (Vel X, Vel Z) in mm/s;
• 95% ellipse;
• Sway area;
• The frequency of COP oscillation in a spectrum [Discrete Fourier Transform of Vector as FFT function in Matlab (R2021)] which implements between 0.1 and 1 Hz: (0.25–0.35 Hz) and (0.35–0.5 Hz) for the medium-low frequencies that account for the vestibular apparatus, and 0.5–0.75 Hz and 0.75–1 Hz for the medium-high frequencies which account for the somatosensory apparatus (24, 30); and
• Root mean square (RMS) in mm as the vector distance from the mean COP to each pair of points in anterior–posterior and medial lateral direction time series (42).

From the side step cutting maneuvers, the following variables were extracted:

• Hip, knee, and ankle joints flexion-extension angles;
• Occurrence of the hip, knee, and ankle peak flexion-extension angles within the sidestep cutting maneuver time frame;
• Hip, knee, and ankle flexion-extension joint angles range of motion (ROM);
• Vertical component of the ground reaction forces;
• Occurrence of the peak force value within the sidestep cutting maneuver time frame;
• Hip, knee, and ankle joints flexion-extension and add-abduction torques; and
• Occurrence of the hip, knee, and ankle peak flexion-extension and add-abduction torques.

Each variable was normalized with respect to the temporal duration of the task considered from the first contact of the landing leg up to the toe off of the opposite leg. All the analyses were performed through self-developed codes in Matlab R2021 (16).

### 2.6. Statistical analysis

Each variable (i.e., posturographic and biomechanical variables) was compared among the different time frames, T1, T2, and T4, through the Friedman test (139 degrees of freedom) for non-parametric repeated measures (Matlab R2021, *p* < 0.05). The Wilcoxon signed rank test (79 degrees of freedom) (Matlab R 2021) was used for comparing variables between T0 and T1 (*p* < 0.05), between EO and EC conditions in each time frame and across the different time frames as follows: T1–T2, T2–T4, and T1–T4. Joint angles, joint torques, and ground reaction forces were also compared at any given instant of time of the cutting maneuver through Friedman and Wilcoxon signed rank tests (*p* < 0.05) (43). Our hypotheses in the three *post-hoc* tests were tested using Bonferroni adjusted alpha levels (0.05/3 = 0.017) using Matlab R 2021. To account for the effect of the device, the effect size (Cohen's *d*) (Supplementary Data B) was calculated (Matlab R2023a) based on the ratio of the difference between group means at couples of different time frames and the pooled standard deviation (44).

## 3. Results

### 3.1. Romberg test

**Figure 3:**
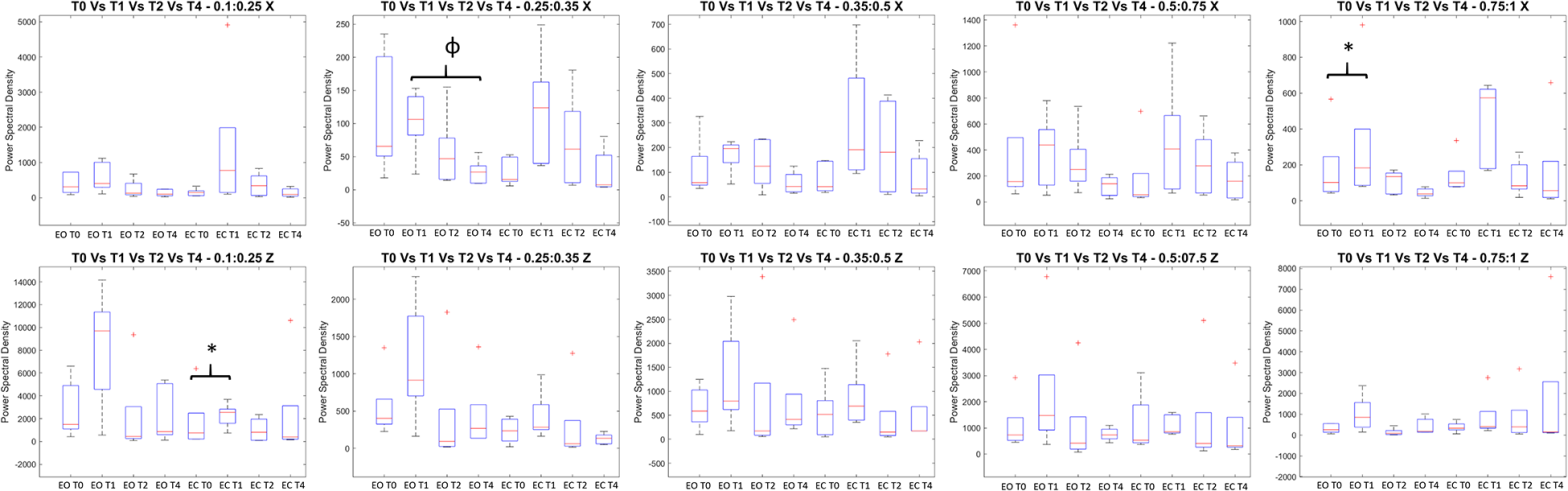
All the variables extracted from the spectral frequencies analysis of the Romberg Test: on the y axis measure units, on the x axis the two conditions (Eyes open EO and Eyes Close EC) for all the analyzed time frames (T0, T1, T2, and T4). The figures report the median value in the middle of the box built with 25% and 75% percentile, while whiskers are upper and lower bounds. Each title reported the frequency range. * = statistically significant difference (p < 0.05 for T0 vs T1, *p* < 0.017 for the others).

**Figure 4:**
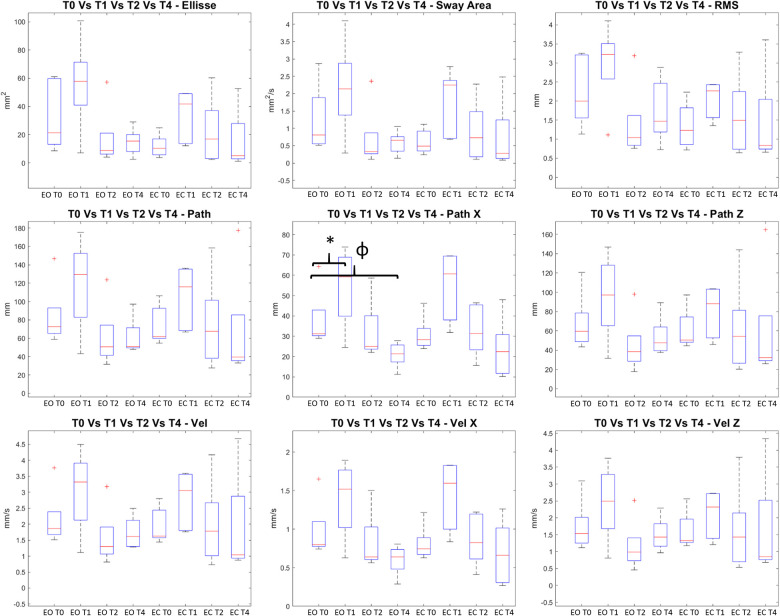
Ellipse, Sway Area, Rms, Path, Path X and Path Z, Velocity, Velocity X and Velocity Z extracted from Romberg Test: on the y axis measure units, on the x axis the two conditions (Eyes open EO and Eyes Close EC) for all the analyzed time frames (T0, T1, T2, and T4). The figures report the median value in the middle of the box built with 25% and 75% percentile, while whiskers are upper and lower bounds. * = statistically significant difference (*p* < 0.05 for T0 vs T1, *p* < 0.017 for the others).

As far as the Romberg test is concerned (see Figures 3, 4, Supplementary Data A1, A2), the Wilcoxon signed rank test revealed a statistically significant increment in both the COP (in the medial lateral direction) and in the frequencies associated with the somatosensory system in EO condition between T1 and T0 and in the frequencies associated with the vestibular system in EC condition. The Friedman test highlighted a statistical significant reduction between T1, T2, and T4 even if these statistical significant differences were not confirmed in the *post-hoc test*s (Supplementary Data A1, A2).

### 3.2. Sidestep cutting maneuver

**Figure 5:**
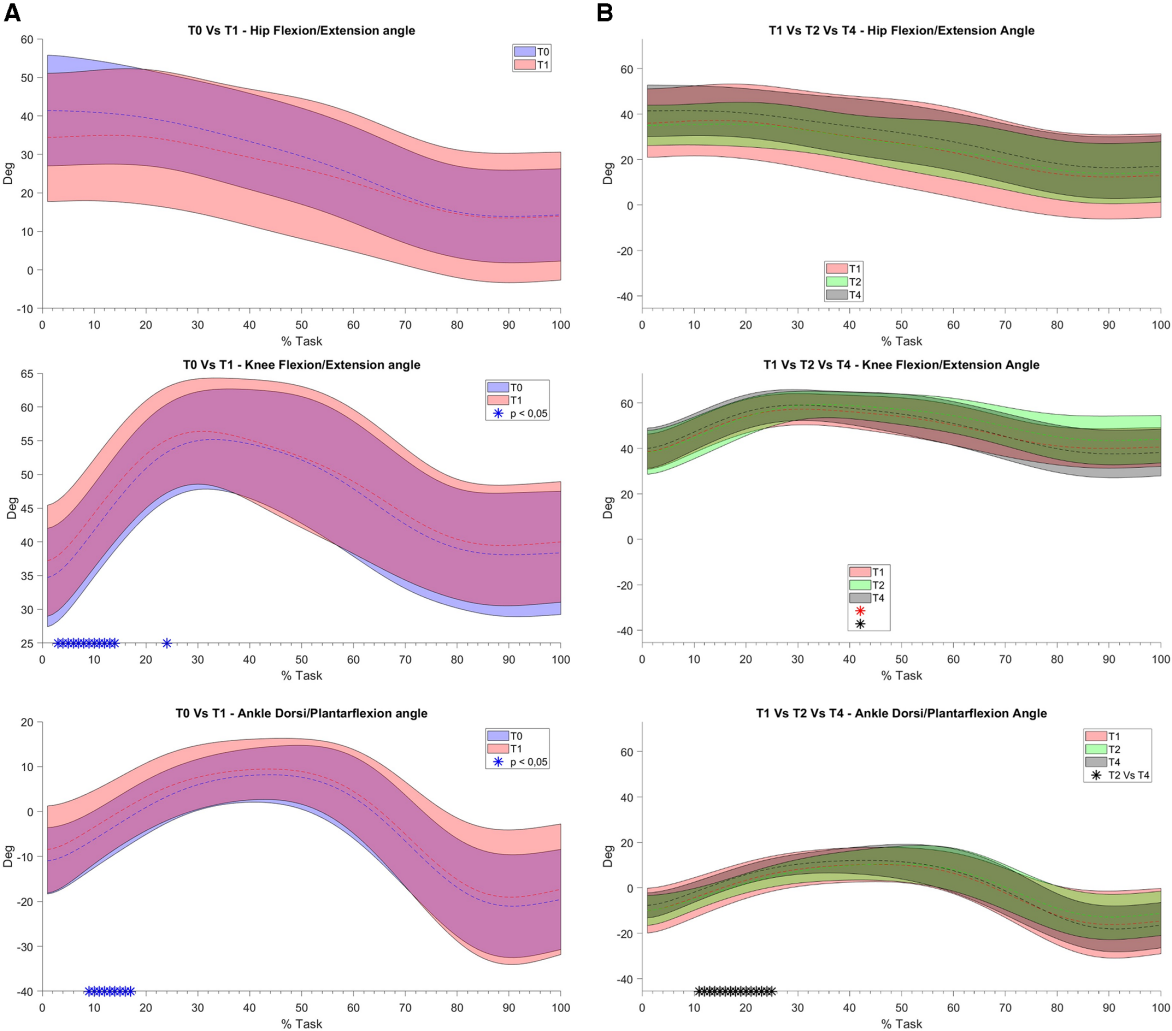
(**A**) and (**B**). Joint angles bands: on the y axis degrees, on the x axis the task normalized on the execution time (0–100%). Mean value (dashed line) ± 1 SD (shaded area). On the (**A**) column the comparison between T0 without Equistasi® (in blue) and T1 with Equistasi® (in red) are reported. * = statistically significant difference (*p* < 0.05) in blue. On the (**B**) column the comparison between T1 (in red) and T2 after two weeks of applications (in green) and T4 after 4 weeks (in grey) are reported. * = statistically significant difference (*p* < 0.017) between T1 and T2 in red, T1 and T4 in green and between T2 and T4 in black.

**Figure 6:**
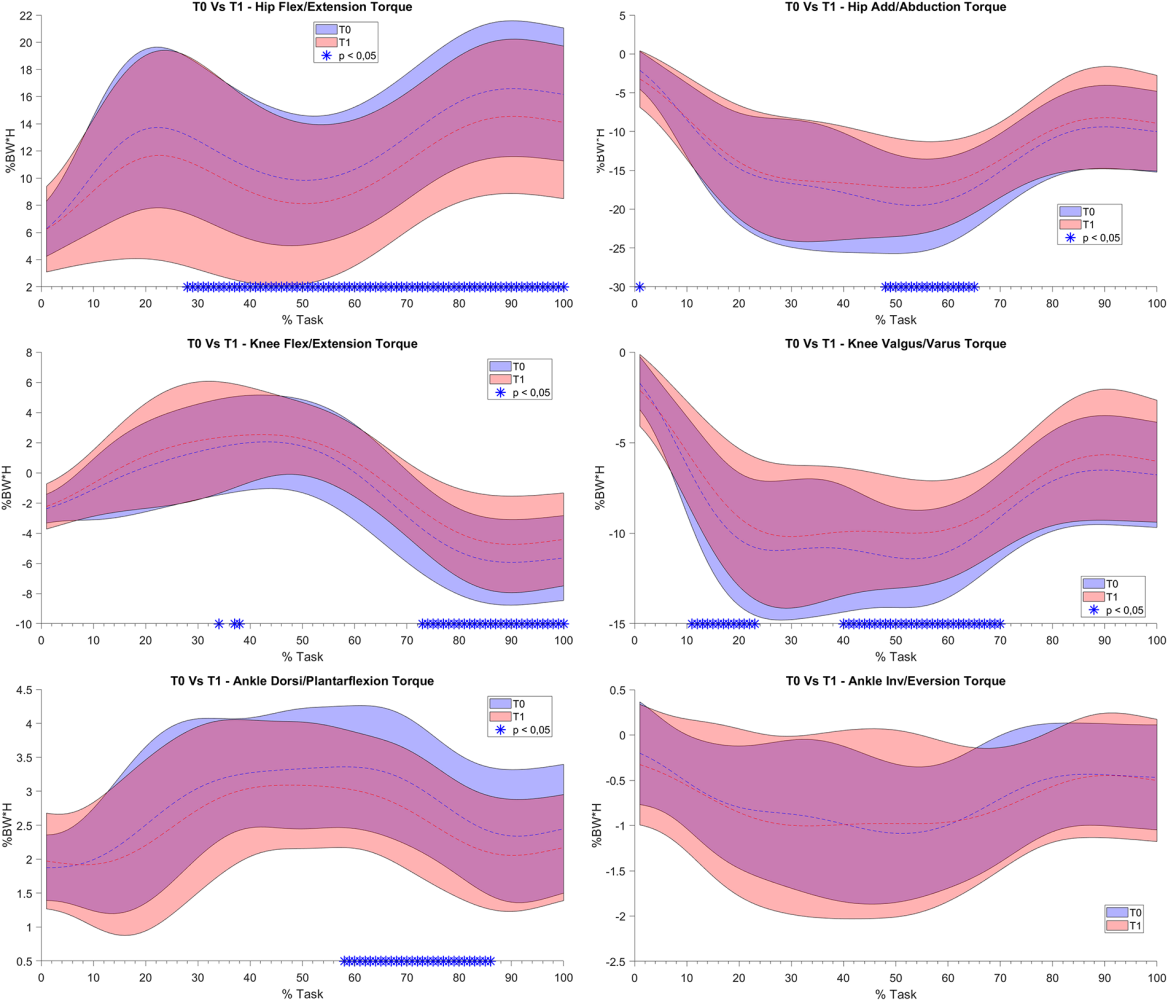
(**A**) and (**B**). Joint torque bands between T0 and T1: on the y axis % Body Weight * Height (%BW*H), on the x axis the task normalized on the execution time (0 -100%). Mean value (dashed line) ± 1 SD (shaded area). T0 without Equistasi® (in blue), T1 with Equistasi® (in red). * = statistically significant difference (*p* < 0.05) between T0 and T1 in blue.

**Figure 7:**
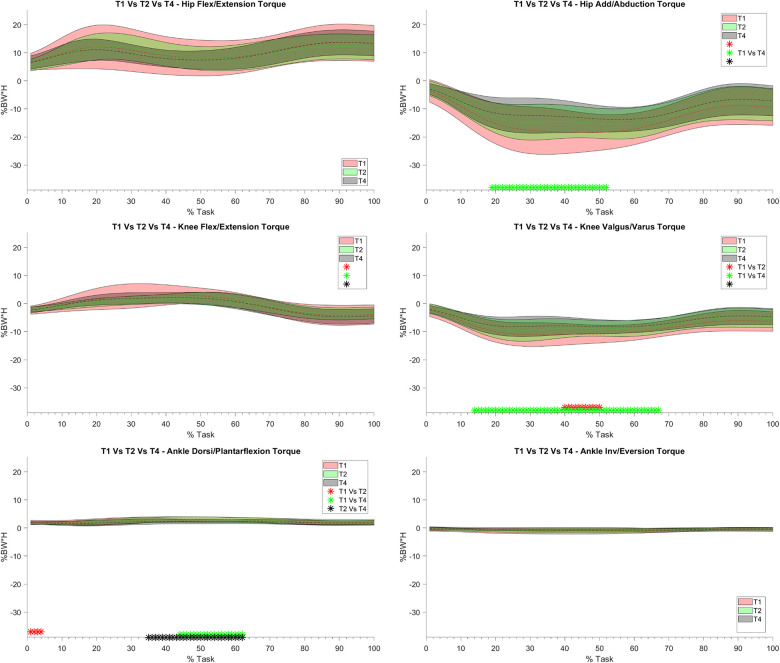
Joint torque bands between T1, T2 and T4: on the y axis % Body Weight * Height (%BW*H), on the x axis the task normalized on the execution time (0 -100%). Mean value (dashed line) ± 1 SD (shaded area). T1 with Equistasi® (in red), T2 after 2 weeks of application (in green), T4 after 4 weeks of application (grey). * = statistically significant difference (*p* < 0.017) between T1 and T2 in red, T1 and T4 in green and between T2 and T4 in black.

**Figure 8:**
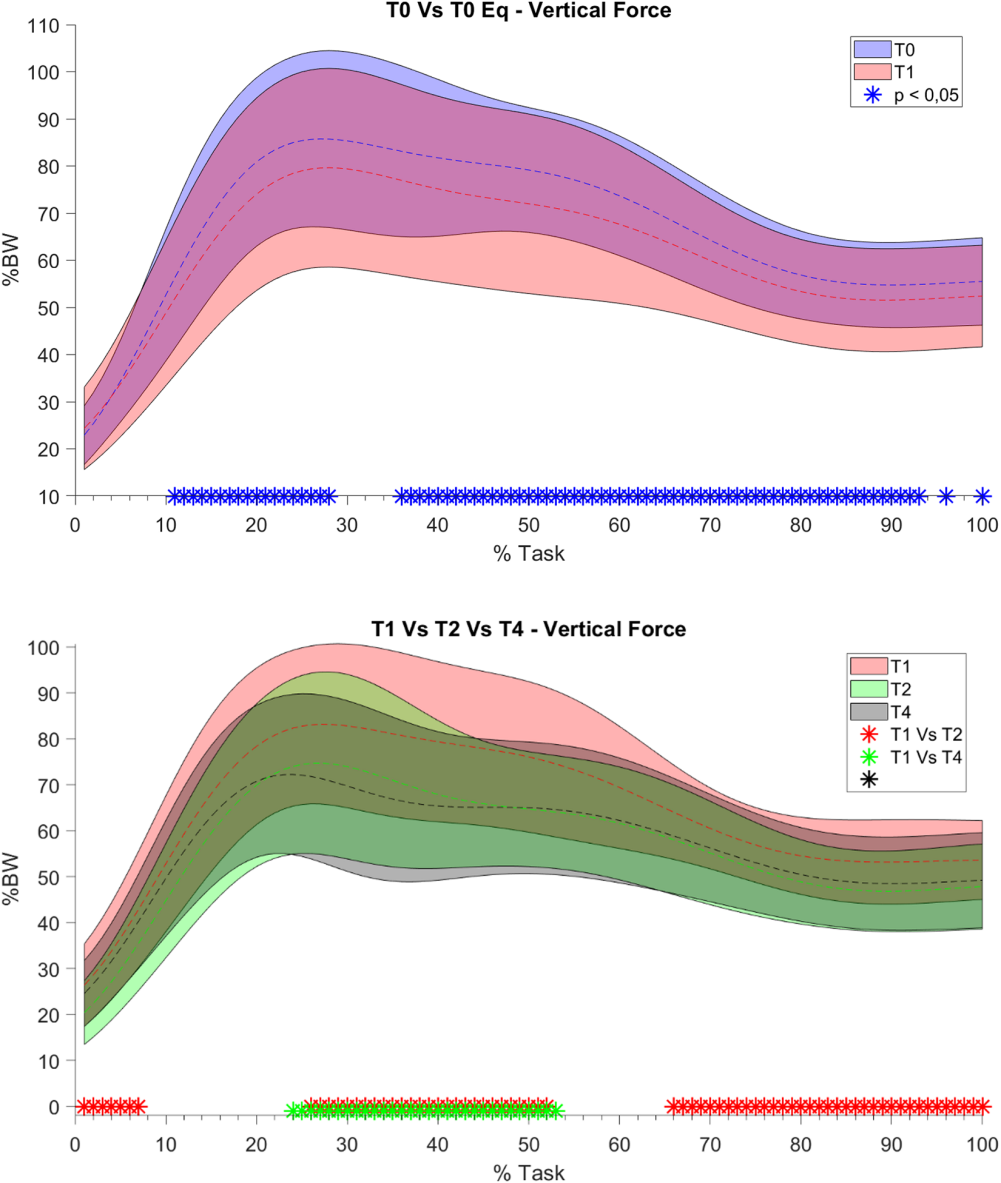
Vertical component of the ground reaction forces. On the y axis % Body Weight, on the x axis the task normalized on the execution time (0 -100%). Mean value (dashed line) ± 1 SD (shaded area). T0 without Equistasi® (in blue), T1 with Equistasi® (in red), T2 after two weeks of applications (in green), T4 after 4 weeks (in grey). * = statistically significant difference (*p* < 0.05 for T0 vs T1, *p* < 0.017 for the others) between T0 and T1 in blue, between T1 and T2 in red, between T1 and T4 in green and between T2 and T4 in black.

**Figure 9:**
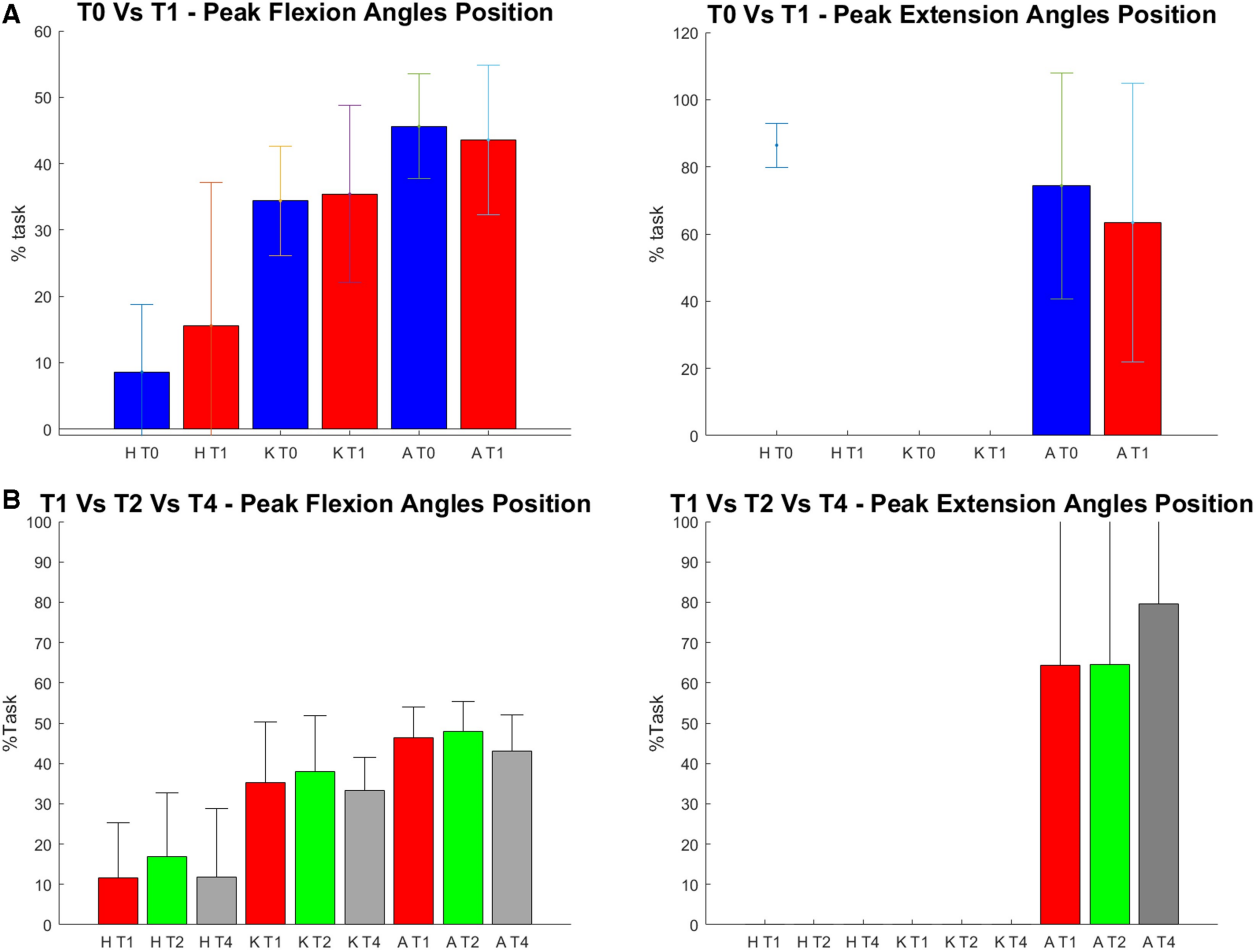
(**A**) and (**B**). Position of the peak of each analyzed angle within the task (in the text referred to as occurrence). On the y axis % task, on the x axis each analyzed time frame (T0, T1, T2 and T4) for each joint (H = Hip, K = Knee, A = Ankle). (**A**) on the top the data of T0 (in blue) and T1 (in red), are reported. * = statistically significant difference (p < 0.05) between the two apex of the parenthesis. (**B**) on the bottom the data of T1 in red, T2 after two weeks of applications in green and T4 after 4 weeks in grey are reported. *= statistically significant difference (*p* < 0.017) between the two apex of the parenthesis.

**Figure 10:**
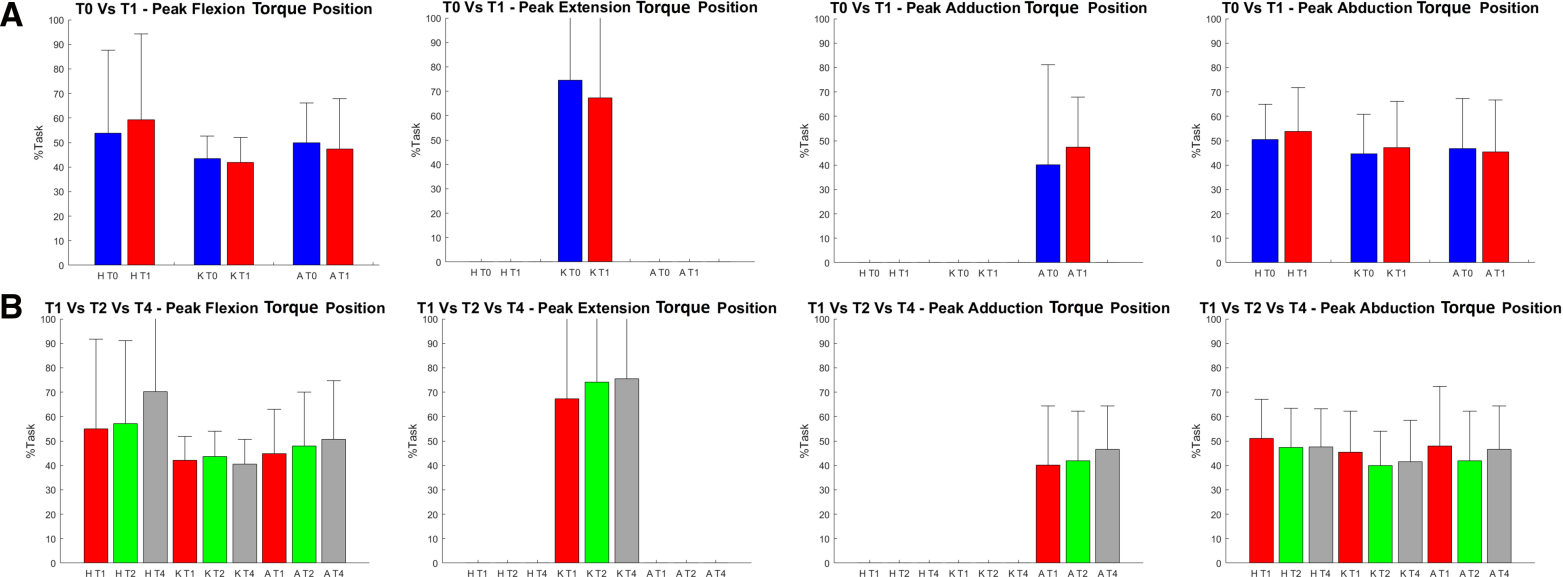
Position of the peak of each analyzed torque within the task (in the text referred to as occurrence). On the y axis % task, on the x axis each analyzed time frame (T0, T1, T2 and T4) for each joint (H = Hip, K = Knee, A = Ankle). (A) on the top it is reported the comparison between T0 (in blue) and T1 (in red ). * = statistically significant difference (*p* < 0.05) between the two apex of the parenthesis. (B) on the bottom the data of T1 (in red), T2 (in green) and T4 (in grey) are reported. * = statistically significant difference (*p* < 0.017 ) between the two apex of the parenthesis.

For what concerns the biomechanical variables, the knee flexion angle was significantly increased between T0 and T1 (see Figure 5). As far as the ankle joint is concerned, a statistically significant reduction in the plantarflexion angle was detected between T0–T1 and T2–T4. When considering the joint torques (see Figures 6, 7), a progressive reduction was detected from T1 up to T4 in both hip and knee abduction torques as well as in the ground reaction forces (see Figure 8). A further reduction in knee and hip flexion-extension torques as well as in hip and knee abduction torques was detected between T0 and T1. Finally, the ankle joint showed a reduction in the dorsiflexion torque between T0 and T1, as well as from T1 up to T4. No statistically significant differences were observed in terms of occurrence of the peak joint angles (Figure 9) and torques (Figure 10).

## 4. Discussion

The main findings of the current study should be considered: the statistically significant reduction observed on the knee abduction torque following the proprioceptive stimulation both after the immediate application of the stimuli (T1) and after 2 and 4 weeks of treatment (T2 and T4, respectively). From a biomechanical point of view, according to Bates et al. (45), this parameter has the potential to generate the greatest change in ACL strain in isolated conditions than other parameters such as anterior tibial shear force or tibial internal rotation. However, in order to assess the overall efficacy of an injury prevention treatment, not only this parameter should be considered (18). In our study, this reduction in knee abduction torque was detected in association with a progressive reduction on hip abduction torque and ground reaction forces from T1 up to T4. A further reduction was noted in the knee flexion-extension angle at T4 in combination with a reduction of the ankle plantarflexion angle. Between T0 and T1 also, lower knee and hip flexion-extension and adduction–abduction torques were detected. Compressively, our results showed the adoption of a landing strategy that complies with the requirement of reducing the biomechanical factors contributing to multiplanar loading (14). In terms of the task chosen for assessing the efficacy of the injury prevention approach, the present study investigated athletes based on the biomechanical analysis of the sidestep cutting maneuver, given the higher rate of incidence of ACL rupture in cutting dominant sports. As the rate is further increased when females are considered rather than males, the study focused on elite female athletes. Given the paucity of research reporting about the efficacy of training programs in terms of biomechanical analysis, an important novelty of the current study should be considered that a complete biomechanical analysis of the lower-limb joints was performed before and after the treatment at different time points. In particular, as no consensus was reached about the most crucial variables for describing cutting maneuvers (33), the ones available for identifying a poor landing strategy were chosen. These are indeed associated with the ligament, quadriceps, trunk, and leg dominance theories developed for landing tasks, namely, hip, knee, and ankle flexion angles (i.e., quadriceps dominance theory); hip, knee, and ankle flexion and adduction–abduction torques (i.e., ligament dominance theory); and vertical ground reaction forces (i.e., softer landings associated with better trunk control) (18). According to the findings of the present study, the proprioceptive stimulation showed to have the potential to improve cutting biomechanics related to the ligament theory, as a decreased knee abduction torque was registered immediately after the device application (T1) and at the two following evaluations (T2 and T4). Noticeably, the observed reduction on hip flexion and abduction torques as well as on hip and knee flexion angles might indicate the achievement of a movement strategy able to overcome the quadriceps dominance theory. Further reduction detected in the vertical ground reaction forces at T1 and T2 suggests an improvement in the ability to perform “softer” contact with the ground. This could be interpreted as an indirect measure of a better trunk control (18). The immediate effects of proprioceptive stimulation can be found in the posturographic analysis results, which showed the promotion of an activity in both the vestibular and somatosensory apparatus and in COP path x (anterior–posterior direction) following the application of the device in T1 respect to T0 (Figure 3 and Supplementary Data A).

These results together with the ones from the Friedman Test are in agreement with recent findings that detected improvements in motor controls of healthy and pathological individuals including athletes (31, 43) after the application of a vibratory stimulus. Even though localized muscle vibration basic principles are known, their effects and the scenario of all possible applications both in sport and clinics are still poorly investigated (46–50). With respect to this, it is important to highlight that the present study adopted localized muscle vibration, which, by targeting directly the muscle–tendon unit of interest, has the advantage of strongly stimulating the muscle spindles and the excitatory drive from the afferent neurons to the homonymous motor unit pool (46–51). This is further supported by a recent work that investigated the effect of Equistasi® device on the somatosensory pathway through the analysis of high-frequency oscillations, and demonstrated that vibrotactile afference, delivered by the device, were able to interfere with the somatosensory processing (35). A number of limitations should be acknowledged: first of all the small sample size does not allow generalization of the current findings and, in particular, this could be the reason why the statistically significant differences highlighted by the Friedman test in the posturographic parameters were not confirmed in the *post-hoc* analysis. The trunk motion was not analyzed; therefore, the potential improvement on the trunk control during high-risk postures was not accounted for. The placebo effect of the application of the device cannot be neglected as the current study lacks a comparison with a placebo device, differently from what was previously performed by the authors in an assessment of a group of individuals with Parkinson's disease (37). The study lacks a comparison with a control group performing a different prevention training, especially the most common ones such as muscle strengthening and stretching. No follow-up was performed in order to define the treatment duration effects; joint kinetics was assessed through plantar pressure data combined with 3D video analysis, and thus possibly affected by similar errors as the one reported in the study by Guiotto et al. (16) when compared with stereophotogrammetry and force plates. However, it should be noted that there is agreement toward encouraging the assessment of the biomechanical risk factors (52) directly on the field, especially when complex gestures are analyzed such as sidestep cutting maneuvers.

In conclusion, the overall study demonstrated the possibility to improve the biomechanics of cutting on a group of female elite soccer players through the application of a vibratory based proprioceptive stimulation with the Equistasi® device. Noticeably, this study assessed the biomechanical risk factors associated with ACL injury risk directly on the field, while performing a task more similar to the sport gestures involved in competition, such as sidestep cutting, and which accounts for the majority of non-contact injuries. Results were encouraging when compared with similar studies reporting about the application of injury prevention programs to improve the biomechanics of landing. Future research is needed to support the current findings, especially by including a larger number of athletes, by adding a placebo device for removing any possible placebo effect, by comparing the treatment with currently used strengthening and stretching programs, and by adding further time point evaluations to detect the duration of the treatment effects.

## Data Availability

The raw data supporting the conclusions of this article will be made available by the authors, without undue reservation.
