# Supplementary material for: Effects of a proprioceptive focal stimulation (Equistasi®) on reducing the biomechanical risk factors associated with ACL injury in female footballers
Source: Front Sports Act Living. 2023 Jul 14;5:1134702. doi: 10.3389/fspor.2023.1134702 (PMC10382620; doi:10.3389/fspor.2023.1134702)
Supplement: Supplementary file 1 [file Datasheet1.pdf]

Supplementary Material

Effects of a proprioceptive focal stimulation (Equistasi®) on reducing the biomechanical risk factors associated with ACL injury in female footballers

Fabiola Spolaor[1*] , Annamaria Guiotto[1†] , Alfredo Ciniglio[1†] , Federica Cibin[2] , Zimi Sawacha[1,3]

[1] Department of Information Engineering, University of Padova, Italy

[2] BBSoF Srl, Padova, Italy

[3] Department of Medicine, University of Padova, Italy

[†] These authors share second authorship

*** Correspondence:**
Corresponding Author

fabiola.spolaor@unipd.it

1 Supplementary Data A

For each variable, Friedman test (Matlab R 2021) was used for the repeated measure non parametric test between T1, T2 and T4 ($p<0.05$). Wilcoxon Signed Rank Test (Matlab R 2021) was used for paired comparison among T0 and T1 ($p<0.05$) and across the different time frames as follows: T1-T2, T2-T4 and T1-T4 ($p<0.017$ after Bonferroni correction). For what concerns joint angles, joint moments, and ground reaction forces the Wilcoxon Signed Rank was applied to compare the variable values at each instant of the cutting maneuver.

All p values available are reported as follows:

Table 1: P values for comparisons on variables Ellipse, Sway Area, Rms, Path, Path X and Path Z, Velocity, Velocity X and Velocity Z extracted from Romberg Test in the two conditions (Eyes open EO and Eyes Close EC). X= medial lateral direction; Z= anterior posterior direction

**Friedman test**	**ellipse**	**sway_area**	**RMS**	**path**	**pathX**	**pathZ**	**velM**	**velMX**	**velMZ**
**EO**	0.074	0.165	0.074	0.247	0.074	0.449	0.165	0.091	0.449
**EC**	0.449	0.549	0.549	0.449	**0.041**	0.449	0.549	0.074	0.549
**Wilcoxon signrank test**	**ellipse**	**sway_area**	**RMS**	**path**	**pathX**	**pathZ**	**velM**	**velMX**	**velMZ**
**EO vs EC**									
**T0**	0.0625	0.0625	0.0625	0.625	0.0625	0.8125	0.625	0.125	0.8125
**T1**	0.125	0.625	0.125	0.3125	1	0.1875	0.3125	1	0.1875
**T2**	0.4375	0.8125	0.3125	0.3125	1	0.1875	0.3125	0.8125	0.125
**T4**	1	1	0.625	0.8125	0.8125	0.8125	1	0.625	0.8125
**EO**									
**T0 vs T1**	0.193	0.084	0.064	0.131	**0.049**	0.322	0.275	0.084	0.375
**T1 vs T2**	0.0625	0.125	0.0625	0.125	0.0625	0.3125	0.125	0.0625	0.3125
**T1 vs T4**	0.125	0.125	0.125	0.125	0.125	0.125	0.125	0.125	0.125

**T2 vs T4**	0.8125	0.625	0.3125	0.8125	0.125	0.3125	0.4375	0.3125	0.1875
**EC**									
**T0 vs T1**	0.232	0.193	0.084	0.160	0.131	0.322	0.232	0.193	0.375
**T1 vs T2**	0.625	0.4375	0.625	0.4375	0.125	0.625	0.4375	0.125	0.625
**T1 vs T4**	0.125	0.125	0.4375	0.3125	0.0625	0.625	0.4375	0.0625	0.625
**T2 vs T4**	0.625	0.8125	1	0.8125	0.1875	1	0.8125	0.625	0.8125

Table 2: P values for comparisons on all the variables extracted from the spectral frequencies analysis of the Romberg Test in the two conditions (Eyes open EO and Eyes Close EC) for all the time frames analyzed (T0,T1,T2, and T4). x= medial lateral direction; z= anterior posterior direction

**Friedman test**	**0_1 0_25_x**	**0_25 0_35_x**	**0_35 0_5_x**	**0_5 0_75_x**	**0_75 1_x**	**0_1 0_25_z**	**0_25 0_35_z**	**0_35 0_5_z**	**0_5 0_75_z**	**0_75 1_z**
**EO**	0.074	0.165	0.165	0.549	0.074	**0.041**	0.165	0.449	0.449	**0.041**
**EC**	**0.041**	0.165	0.247	0.449	0.074	0.074	0.165	**0.007**	0.247	0.247
**Wilcoxon signrank test**	**0_1 0_25_x**	**0_25 0_35_x**	**0_35 0_5_x**	**0_5 0_75_x**	**0_75 1_x**	**0_1 0_25_z**	**0_25 0_35_z**	**0_35 0_5_z**	**0_5 0_75_z**	**0_75 1_z**
**EO vs EC**										
**T0**	0.0625	0.0625	0.4375	0.0625	1	0.4375	0.125	0.8125	0.625	1
**T1**	0.3125	0.8125	0.4375	1	0.3125	0.125	0.3125	1	0.3125	0.625
**T2**	1	1	0.625	1	0.625	1	0.4375	0.4375	0.4375	0.0625
**T4**	1	1	0.8125	0.4375	0.625	0.8125	0.1875	0.0625	0.8125	0.625
**EO**										
**T0 vs T1**	0.322	1.000	0.232	1.000	**0.002**	0.557	0.770	0.492	0.770	0.846
**T1 vs T2**	0.3125	0.1875	0.4375	1	0.4375	0.0625	0.1875	0.4375	0.625	0.0625
**T1 vs T4**	0.0625	0.125	0.0625	0.125	0.0625	0.125	0.125	0.625	0.1875	0.1875
**T2 vs T4**	0.625	0.1875	0.3125	0.3125	0.3125	0.4375	0.625	0.8125	0.625	0.125
**EC**										

**T0 vs T1**	0.193	0.232	0.084	0.922	0.131	**0.049**	0.625	0.131	1.000	0.432
**T1 vs T2**	0.4375	0.625	0.625	0.625	0.0625	0.0625	0.4375	0.0625	0.625	0.625
**T1 vs T4**	0.0625	0.0625	0.125	0.1875	0.125	0.625	0.125	0.0625	0.625	0.625
**T2 vs T4**	0.1875	0.4375	0.1875	0.625	0.625	0.8125	0.625	0.0625	0.8125	0.4375

Table 3: P values for comparisons on position of the peak of each angle analyzed within the task for each joint (H=Hip, K= Knee, A= Ankle).

	**PosMax Hip FEAngle**	**PosMax Knee FEAngle**	**PosMax Ankle FEAngle**	**PosMin Hip FEAngle**	**PosMin Knee FEAngle**	**PosMin Ankle FEAngle**
**T0 vs T1 - Wilcoxon signrank**	0.872	0.808	0.310	0.288	0.913	0.935
**T1-T2-T4 - Friedman**	0.126	0.268	0.052	0.411	0.023	0.160
**T1 vs T2 - Wilcoxon signrank**	0.314	0.315	0.353	0.930	0.628	0.815
**T1 vs T4 - Wilcoxon signrank**	0.698	0.939	0.083	0.173	0.056	0.061
**T2 vs T4 - Wilcoxon signrank**	0.135	0.130	0.010	0.210	0.008	0.023

Table 4: P values for comparisons on position of the peak of each moment analyzed within the task for each joint (H=Hip, K= Knee, A= Ankle).

	PosMax Hip FETorque	PosMax Hip AATorque	PosMax Knee FETorque	PosMax Knee VVTorque	PosMax Ankle FETorque	PosMax Ankle IETorque
**T0 vs T1 - Wilcoxon signrank**	0.754	0.230	0.782	0.781	0.253	0.155
**T1-T2-T4 - Friedman**	0.216	0.513	0.241	0.789	0.460	0.278
**T1 vs T2 - Wilcoxon signrank**	0.779	0.507	0.457	1.000	0.407	0.149
**T1 vs T4 - Wilcoxon signrank**	0.071	0.211	0.603	1.000	0.273	0.092
**T2 vs T4 - Wilcoxon signrank**	0.281	0.594	0.106	0.844	0.891	0.905
	**PosMinHip FETorque**	**PosMinHip AATorque**	**PosMinKnee FETorque**	**PosMinKnee VVTorque**	**PosMinAnkle FETorque**	**PosMinAnkle IETorque**
**T0 vs T1 - Wilcoxon signrank**	0.542	0.808	0.573	0.661	0.765	0.647
**T1-T2-T4 - Friedman**	0.097	0.964	0.355	0.359	0.006	0.308
**T1 vs T2 - Wilcoxon signrank**	0.154	0.456	0.476	0.249	0.097	0.420
**T1 vs T4 - Wilcoxon signrank**	0.397	0.819	0.155	0.523	0.638	0.931
**T2 vs T4 - Wilcoxon signrank**	0.048	0.795	0.263	0.658	0.002	0.214

Table 5: P values for comparisons on vertical component of the ground reaction forces

	PosMax VerticalForce
**T0 vs T1 - Wilcoxon signrank**	0.423
**T1-T2-T4 - Friedman**	0.268
**T1 vs T2 - Wilcoxon signrank**	0.483
**T1 vs T4 - Wilcoxon signrank**	0.130
**T2 vs T4 - Wilcoxon signrank**	0.079

2 Supplementary data B

To account for the effect of the device, the effect size (Cohen's d) was calculated (Matlab R2023a) based on the ratio of the difference between group means at couples of different T and the pooled standard deviation (Cohen J. 1988. Statistical power analysis for the behavioral sciences. 2nd ed. New York (NY): Lawrence Erlbaum Associates). Ref 44 in the main text.

Table 1. The effect size (Cohen's d) for the position of the peak of each angle and each torque analyzed within the task for each joint .

	T0 vs T1	T1 vs T2	T1 vs T4	T2 vs T4
**Peak flexion angle position - Hip**	-0.2087	-0.3482	-0.0209	0.2959
**Peak flexion angle position - Knee**	-0.1585	-0.1784	0.1540	0.3894

**Peak flexion angle position - Ankle**	-0.3013	-0.2115	0.3698	0.5712
**Peak extension angle position - Hip**	-0.1984	0.3312	0.0622	-0.2389
**Peak extension angle position - Knee**	-0.0921	0.2273	-0.5270	-0.7718
**Peak extension angle position - Ankle**	0.1746	-0.0078	-0.4054	-0.4012
**Peak flexion torque position - Hip**	-0.0339	-0.0615	-0.4261	-0.3794
**Peak adduction torque position - Hip**	-0.3667	0.3566	0.3653	0.0071
**Peak flexion torque position - Knee**	-0.1358	-0.1360	0.1621	0.2881
**Peak valgus torque position - Knee**	-0.0988	0.2012	0.0873	-0.1127
**Peak flexion torque position - Ankle**	0.4185	-0.1470	-0.2640	-0.1150
**Peak inversion torque position - Ankle**	-0.2439	0.3280	0.2984	-0.0244
**Peak extension torque position - Hip**	-0.1357	0.3154	-0.2311	-0.5356
**Peak abduction torque position - Hip**	-0.1207	0.2309	0.2194	-0.0152
**Peak extension torque position - Knee**	0.1325	-0.1806	-0.2162	-0.0391
**Peak varus torque position - Knee**	-0.0598	0.3512	0.2295	-0.1003
**Peak extension torque position - Ankle**	-0.1392	0.3616	-0.1678	-0.5462
**Peak eversion torque position - Ankle**	0.1273	0.2638	0.0679	-0.2328

Table 7. The effect size (Cohen’s d) for the Ellipse, Sway Area, Rms, Path, Path X and Path Z, Velocity, Velocity X and Velocity Z extracted from Romberg Test and for all the variables extracted from the spectral frequencies analysis of the Romberg Test in the two conditions (Eyes open EO and Eyes Close EC). X= medial lateral direction; Z= anterior posterior direction

	**EO T0 Vs EC T0**	**EO T1 Vs EC T1**	**EO T2 Vs EC T2**	**EO T4 Vs EC T4**	**EO T0 Vs EO T1**	**EC T0 Vs EC T1**	**EO T1 Vs EO T2**	**EO T1 Vs EO T4**	**EC T1 Vs EC T2**	**EC T1 Vs EC T4**	**EO T2 Vs EO T4**	**EC T2 Vs EC T4**
**Ellisse**	0.894	0.670	-0.179	-0.077	-0.617	-1.179	1.085	1.336	0.401	0.667	0.112	0.212
**Sway Area**	0.692	0.277	-0.183	-0.191	-0.591	-1.221	0.986	1.248	0.713	0.785	0.128	0.119
**RMS**	0.908	0.871	-0.175	0.205	-0.549	-0.957	1.181	0.977	0.390	0.462	-0.308	0.097
**Path**	0.262	0.244	-0.251	-0.112	-0.615	-0.831	1.020	1.159	0.531	0.580	0.000	0.104
**Path X**	0.478	-0.014	-0.009	-0.181	-0.723	-1.354	0.963	1.802	1.110	1.506	0.810	0.527
**Path Z**	0.182	0.342	-0.315	-0.104	-0.569	-0.568	1.038	0.944	0.354	0.335	-0.263	0.026
**Vel**	0.208	0.192	-0.282	-0.150	-0.615	-0.831	1.020	1.041	0.531	0.479	-0.164	0.014
**Vel X**	0.422	-0.075	-0.057	-0.219	-0.723	-1.354	0.963	1.632	1.110	1.345	0.621	0.367
**Vel Z**	0.132	0.295	-0.340	-0.139	-0.569	-0.568	1.038	0.837	0.354	0.243	-0.422	-0.053
**0.1:0.25 X**	0.890	-0.441	-0.303	-0.058	-0.402	-0.701	0.763	1.160	0.571	0.702	0.475	0.699
**0.25:0.35 X**	1.024	-0.141	-0.181	-0.043	0.104	-1.131	0.703	1.646	0.453	1.080	0.569	0.626
**0.35:0.5 X**	0.336	-0.568	-0.357	-0.278	-0.428	-0.973	0.329	1.542	0.355	0.910	0.793	0.630
**0.5:0.75 X**	0.377	-0.157	0.035	-0.333	0.007	-0.577	0.216	0.979	0.342	0.653	0.791	0.466
**0.75:1 X**	0.187	-0.324	-0.199	-0.482	-0.325	-1.266	0.591	0.777	1.351	0.837	0.981	-0.146
**0.1:0.25 Z**	0.334	1.257	0.324	0.004	-1.040	-0.210	1.024	1.125	0.940	-0.029	-0.046	-0.326
**0.25:0.35 Z**	0.736	0.954	0.141	0.685	-0.752	-0.600	0.758	0.851	0.251	1.033	-0.041	0.334
**0.35:0.5 Z**	0.188	0.367	0.264	0.196	-0.593	-0.405	0.288	0.398	0.467	0.328	0.035	-0.108
**0.5:07.5 Z**	-0.056	0.523	-0.074	-0.190	-0.487	0.079	0.439	0.677	-0.090	0.079	0.210	0.121
**0.75:1 Z**	-0.224	0.130	-0.616	-0.458	-0.896	-0.503	1.127	0.706	0.004	-0.296	-0.749	-0.291

Figure 8. The effect size (Cohen’s d) for angles and torques for time series (bands)

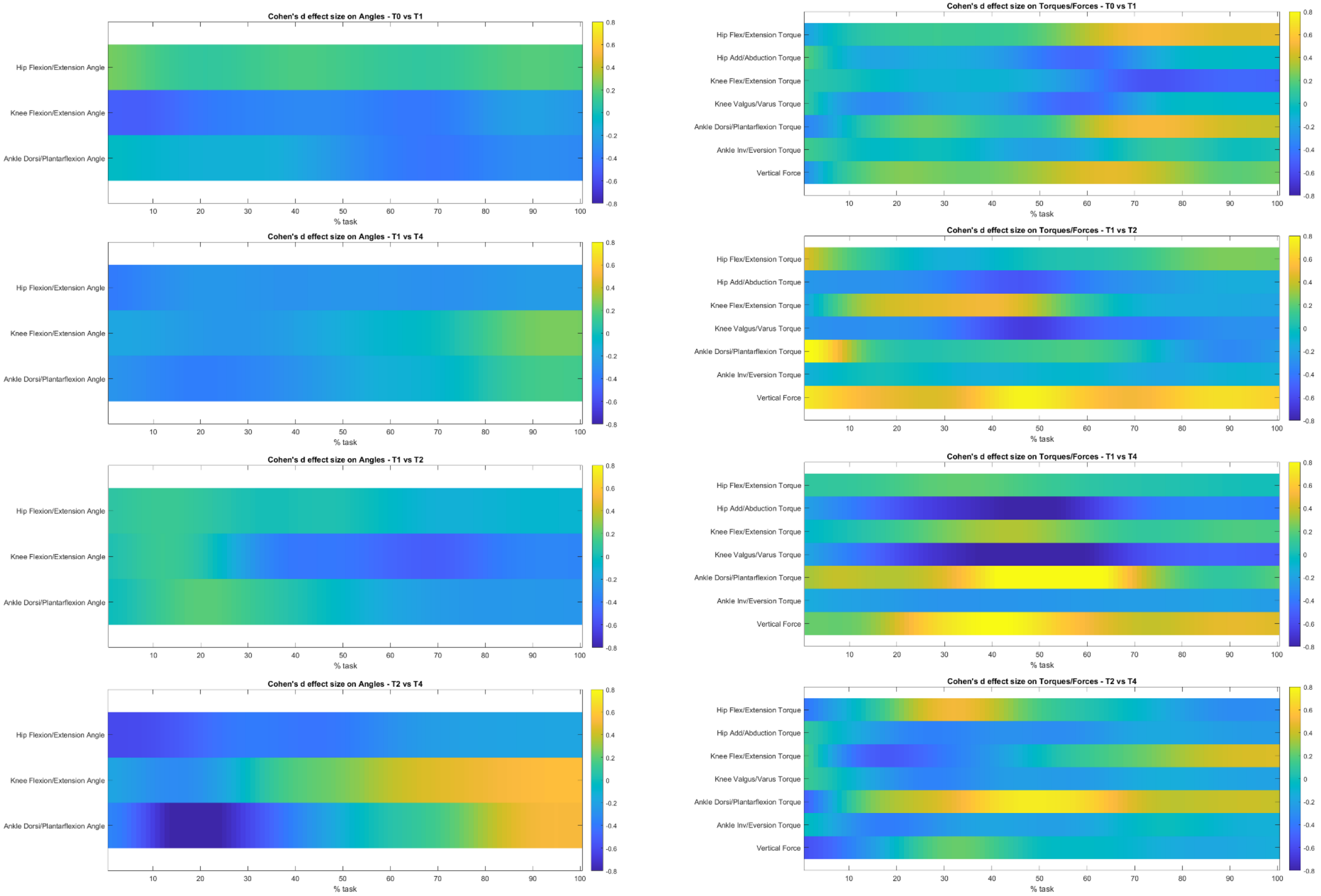

Cohen's d effect size on Angles - T0 vs T1
Hip Flexion/Extension Angle
Knee Flexion/Extension Angle
Ankle Dorsi/Plantarflexion Angle
% task
Cohen's d effect size on Angles - T1 vs T4
Hip Flexion/Extension Angle
Knee Flexion/Extension Angle
Ankle Dorsi/Plantarflexion Angle
% task
Cohen's d effect size on Angles - T1 vs T2
Hip Flexion/Extension Angle
Knee Flexion/Extension Angle
Ankle Dorsi/Plantarflexion Angle
% task
Cohen's d effect size on Angles - T2 vs T4
Hip Flexion/Extension Angle
Knee Flexion/Extension Angle
Ankle Dorsi/Plantarflexion Angle
% task
Cohen's d effect size on Torques/Forces - T0 vs T1
Hip Flex/Extension Torque
Hip Add/Abduction Torque
Knee Flex/Extension Torque
Knee Valgus/Varus Torque
Ankle Dorsi/Plantarflexion Torque
Ankle Inv/Eversion Torque
Vertical Force
% task
Cohen's d effect size on Torques/Forces - T1 vs T2
Hip Flex/Extension Torque
Hip Add/Abduction Torque
Knee Flex/Extension Torque
Knee Valgus/Varus Torque
Ankle Dorsi/Plantarflexion Torque
Ankle Inv/Eversion Torque
Vertical Force
% task
Cohen's d effect size on Torques/Forces - T1 vs T4
Hip Flex/Extension Torque
Hip Add/Abduction Torque
Knee Flex/Extension Torque
Knee Valgus/Varus Torque
Ankle Dorsi/Plantarflexion Torque
Ankle Inv/Eversion Torque
Vertical Force
% task
Cohen's d effect size on Torques/Forces - T2 vs T4
Hip Flex/Extension Torque
Hip Add/Abduction Torque
Knee Flex/Extension Torque
Knee Valgus/Varus Torque
Ankle Dorsi/Plantarflexion Torque
Ankle Inv/Eversion Torque
Vertical Force
% task
